# Nerve Ultrasound Detects Peripheral Nerve Enlargement in Cerebrotendinous Xanthomatosis

**DOI:** 10.1002/mus.70221

**Published:** 2026-03-26

**Authors:** Antonio Edvan Camelo‐Filho, Pedro Lucas G. S. B. Lima, Tito B. S. Soares, Rodrigo Fagundes da Rosa, Luis Edmundo T. A. Furtado, Ana Lucila Moreira, André L. S. Pessoa, Paulo R. Nóbrega, Pedro Braga‐Neto

**Affiliations:** ^1^ Division of Neurology, Department of Clinical Medicine Universidade Federal do Ceará Fortaleza Ceará Brazil; ^2^ Center of Health Sciences Universidade Estadual do Ceará Fortaleza Ceará Brazil; ^3^ Department of Neurology Centro Universitário UNINTA Sobral Ceará Brazil; ^4^ Department of Neurology, Faculdade de Medicina Universidade de São Paulo São Paulo Brazil; ^5^ Albert Sabin Hospital Fortaleza Ceará Brazil

**Keywords:** cerebrotendinous xanthomatosis, hereditary neuropathies, nerve ultrasound, peripheral neuropathy, tendon xanthomas

## Abstract

**Introduction/Aims:**

Cerebrotendinous xanthomatosis (CTX) is a rare autosomal recessive disorder caused by variants in the *CYP27A1* gene, resulting in cholestanol accumulation in various tissues, including peripheral nerves. Polyneuropathy is common but often under‐recognized in CTX. This study aimed to evaluate the cross‐sectional area (CSA) of peripheral nerves in CTX.

**Methods:**

Six genetically confirmed CTX patients underwent clinical, electrophysiological, and ultrasonographic evaluations. Clinical severity was assessed using the Scale for the Assessment and Rating of Ataxia (SARA) and the Polyneuropathy Disability (PND) score. Nerve ultrasound was performed at standardized sites of the median, ulnar, tibial, and sural nerves, and at the supraclavicular brachial plexus. CSA values were compared with healthy controls.

**Results:**

Nerve conduction studies (NCS) identified demyelinating polyneuropathy in three patients. However, ultrasound showed nerve enlargement in all six, including those with normal NCS. The supraclavicular brachial plexus was enlarged in every patient. In 5 adult patients, enlargement was most consistently observed in the median nerve (4 at the wrist and forearm; 5 at the cubital fossa and upper arm), the ulnar nerve at the upper arm (5), and the fibular nerve at the fibular head (4).

**Discussion:**

This study demonstrates that nerve ultrasound can detect peripheral nerve enlargement in CTX, even in preclinical stages of polyneuropathy. Enlargement of peripheral nerves may be a sensitive marker of disease severity and peripheral nerve involvement. The role of nerve ultrasound as a diagnostic tool in CTX is promising, and future longitudinal studies are needed to determine its value in disease monitoring.

**Abbreviations**
ARSACS: Autosomal recessive spastic ataxia of charlevoix–saguenay
A‐T: Ataxia–telangiectasia
ATTRv: hereditary transthyretin amyloidosis
BMI: Body mass index
CANVAS: Cerebellar ataxia with neuropathy and vestibular areflexia syndrome
CDCA: chenodeoxycholic acid
CIDP: chronic inflammatory demyelinating polyneuropathy
CSA: cross‐sectional area
CTX: cerebrotendinous xanthomatosis
EMG: electromyography
MHz: megahertz
MLD: Metachromatic leukodystrophy
MRN: Magnetic resonance neurography
NCS: nerve conduction studies
NCV: nerve conduction velocity
NMUS: Neuromuscular ultrasound
PND: polyneuropathy disability
SARA: Scale for the Assessment and Rating of Ataxia
SCA2: Spinocerebellar Ataxia Type 2
X‐ALD: XLinked Adrenoleukodystrophy

## 1 Introduction

Cerebrotendinous xanthomatosis (CTX) is a rare autosomal recessive lipid storage disorder caused by pathogenic variants in the *CYP27A1* gene, which encodes the sterol 27‐hydroxylase, an enzyme in the cytochrome P450 oxidase family [1]. This enzyme converts cholesterol to bile acids, and its compromise leads to decreased bile acid synthesis and an increase in the production of cholestanol, which accumulates in body tissues, particularly in tendons, eyes, and the peripheral and central nervous systems [2, 3, 4, 5].

CTX has increasingly been recognized as an underdiagnosed disorder. Although classified as rare [6], CTX exhibits striking variability in prevalence among different populations [4]. The prevalence among individuals of Sephardi and Ashkenazi Jewish descent is relatively high, with estimates reaching approximately 1 in 108 in some series [7, 8], whereas in Brazil, the estimated prevalence is about 0.64 cases per million inhabitants [9], consistent with global estimates of roughly 1 per million [6]. This under‐recognition is likely related to the disease's highly heterogeneous clinical presentation, which encompasses a broad spectrum of manifestations, severities, and ages of onset [9, 10].

Peripheral neuropathy is a frequently reported and disabling feature of CTX [11, 12, 13, 14]. Nerve conduction studies and electromyography (NCS and EMG) may reveal findings consistent with demyelinating, axonal, or mixed forms of polyneuropathy [13, 15]. Signs and symptoms of polyneuropathy are often subtle or difficult to detect because central nervous system and psychiatric involvement may overshadow the clinical presentation [16]. The severity of polyneuropathy varies substantially among patients, ranging from asymptomatic to severe [17]. Assessing the peripheral nervous system may play a role in the diagnosis and the ongoing monitoring of CTX [18].

Peripheral nerve ultrasound is a valuable tool for diagnosing hereditary neuropathies [19]. A few studies have reported ultrasound evidence of nerve enlargement in patients with CTX, highlighting its potential as a non‐invasive method for further characterizing peripheral nerve involvement [20, 21]. This study aims to characterize the patterns and extent of peripheral nerve abnormalities in CTX patients with a primary focus on peripheral nerve ultrasound evaluation supported by clinical assessment and complementary NCS and EMG findings.

## 2 Methods

### 2.1 Participants

This was an observational cross‐sectional study that prospectively included six CTX patients from our outpatient clinic for ataxias between July 2024 and March 2025. All patients had a confirmed genetic diagnosis of CTX and underwent NCS/EMG and nerve ultrasound. Information regarding disease duration, age at symptom onset, cataracts, and xanthomas was collected using a standardized form. The clinical assessment included a standardized neurological examination incorporating the scale for the assessment and rating of ataxia (SARA) [22] and the polyneuropathy disability (PND) score [23]. The SARA quantifies the severity of ataxia, with scores ranging from 0 (no ataxia) to 40 (most severe), based on evaluations of gait, stance, coordination, and speech [22]. The PND scale, originally developed for transthyretin amyloidosis with polyneuropathy (ATTRv), provides an ordinal classification of walking ability, ranging from complete independence to total immobility [23]. It consists of five stages: PND I, sensory symptoms without gait disturbance; PND II, need for support when walking but no assistive devices; PND IIIa and IIIb, requirement of one or two sticks, respectively; PND IV, wheelchair dependence; and PND V, bedridden status [23].

For the nerve ultrasound control group, six healthy age‐, sex‐, and body mass index (BMI)‐matched controls were recruited from the medical staff. The study was approved by the Ethics Committee of the Federal University of Ceará under protocol number 6.945.762. Informed consent was obtained from all participants, including from the legal guardian of the minor participant.

### 2.2 Ultrasound and Electrophysiology

B‐mode nerve ultrasound protocol was performed by a physician experienced in neuromuscular ultrasound (A.E.C.‐F.) using a high‐resolution linear probe (6–15 MHz, LOGIQ 9, GE Healthcare, Chicago, IL, USA) to assess peripheral nerves on the right side of the body. Median and ulnar nerves were examined at the wrist, mid‐forearm, elbow and mid‐humerus; sural nerve at the distal calf; tibial nerve at the popliteal fossa and ankle, and brachial plexus at the supraclavicular level. The cross‐sectional area (CSA) of the brachial plexus was measured in the supraclavicular space at the level of the divisions—distal to the trunks and proximal to the cords—following the methodological approach described by Salvalaggio et al. [24]. To improve anatomical delineation and minimize inclusion of non‐neural tissue, power Doppler imaging was systematically applied to identify and exclude vascular structures. Three CSA measurements were made at each site using the trace function on the ultrasound device, and the mean value was determined.

Additionally, CTX patients underwent NCS and EMG tests performed under standard conditions using a Litebox workstation (Neurosoft, Ivanovo, Russia), which included motor (median, ulnar, fibular, and tibial) and antidromic sensory (median, ulnar, and sural) conduction studies. Polyneuropathy was classified as demyelinating when nerve conduction velocity (NCV) fell below 75% of the lower limit of normal or when distal latency exceeded 130% of the upper limit of normal [25]. An axonal pattern was defined by markedly reduced response amplitudes, accompanied by only mild slowing of the NCV or prolongation of the distal latency. These criteria are consistent with international guidelines for the electrophysiological evaluation of chronic inflammatory demyelinating polyneuropathy (CIDP) [25].

### 2.3 Statistics

Due to the small sample size and the exploratory nature of this study, formal statistical analyses were not conducted. Nerve CSA in CTX patients was reported as absolute values in mm^2^ and compared with matched controls and reference values [24, 26] (brachial plexus at the supraclavicular level ≤ 82 mm^2^; median nerve at the wrist ≤ 10 mm^2^, forearm ≤ 9 mm^2^, elbow ≤ 12 mm^2^, and arm ≤ 12 mm^2^; ulnar nerve at the wrist ≤ 9 mm^2^, forearm ≤ 8 mm^2^, elbow ≤ 10 mm^2^, and arm ≤ 8 mm^2^; fibular nerve at the fibular head ≤ 12 mm^2^ and popliteal fossa ≤ 10 mm^2^; sural nerve ≤ 3 mm^2^; and tibial nerve at the popliteal fossa ≤ 32 mm^2^ and ankle ≤ 12 mm^2^).

## 3 Results

This study included six patients with CTX, three of whom were male. Table 1 summarizes the clinical profile of patients. The median age at evaluation was 33.5 years (range: 9–46), and the mean disease duration was 20.5 years (range: 9–31). Symptoms started mainly in childhood and adolescence, and most patients developed psychiatric and neurological symptoms with major variation in additional clinical features. Parental consanguinity was reported in three out of six patients. All patients experienced a substantial diagnostic delay, with a mean interval of 14.8 years (range: 6–31 years) from symptom onset to diagnosis. Three patients (pts 1, 3, and 6) in our cohort were on chenodeoxycholic acid (CDCA) at a standard dose of 15 mg/kg/day, divided into three daily administrations, for 2 years before evaluation. Five patients presented with cerebellar ataxia. Other common findings included chronic diarrhea in three patients, cataracts in three, and pyramidal signs in four.

**TABLE 1:** Clinical, demographic, and genetic characteristics of patients with cerebrotendinous xanthomatosis.

	Patient 1	Patient 2	Patient 3	Patient 4	Patient 5	Patient 6
Age at evaluation (y)	9	34	27	31	46	40
Age of diagnosis (y)	6	29	25	31	43	39
Disease duration (y)	9	16	12	31	31	25
Sex	F	F	M	F	M	M
BMI (kg/m^2^)	14.1	25.9	18.3	21.2	27.5	26.4
First symptom	Neonatal cholestasis	Tendon xanthomas	Cataract	Neonatal cholestasis	Psychiatric disturbance	Tendon xanthomas
Number of xanthomas	0	5	2	4	5	4
SARA	0	2	3	8	22	25.5
PND	0	0	0	1	2	3a
Polyneuropathy	—	—	—	Demyelinating	Demyelinating	Demyelinating
Colesthanol	High	High	N/A	N/A	High	High
Brain MRI	Abnormal	Abnormal	Normal	Abnormal	Abnormal	Abnormal
CDCA use	Yes	Yes	No	No	No	Yes
Variant 1	c.1183C > T (p.Arg395Cys)	c.886C > T (p.Gln296*)	c.1183C > T (p.Arg395Cys)	c.379C > T (p.Arg127Trp)	c.1183C > T (p.Arg395Cys)	c.1181T> C (p.Leu394Pro)
Variant 2	c.1183C > T (p.Arg395Cys)	c.1421G > A (p.Arg474Gln)	c.1183C > T (p.Arg395Cys)	c.379C > T (p.Arg127Trp)	c.1183C > T (p.Arg395Cys)	c.1181T > C (p.Leu394Pro)
Other clinical features	Intellectual disability psychiatric symptoms pyramidal signs chronic diarrhea	Psychiatric symptoms pyramidal signs chronic diarrhea	Cataracts psychiatric symptoms	Cataracts psychiatric symptoms cognitive decline chronic diarrhea	Cataracts psychiatric symptoms pyramidal signs epilepsy *Pes cavus*	Cataracts psychiatric symptoms cognitive decline pyramidal signs epilepsy *Pes cavus*

*Note*: * denotes a nonsense variant.

Abbreviations: BMI = Body mass index; CDCA = Chenodeoxycholic acid; MRI = Magnetic resonance imaging; N/A = Not available; PND = Polyneuropathy Disability score; SARA = Scale for the Assessment and Rating of Ataxia.

Demyelinating polyneuropathy was identified in 3 patients (pts. 4, 5, and 6), and characterized by reduced motor conduction velocities, temporal dispersion, and prolonged or absent F‐wave latencies (see Table S1). Causes for acquired polyneuropathy were excluded in all participants. Polyneuropathy patients had higher PND scores (2–3a), whereas the remaining three patients with normal NCS (pts 1–3) showed no clinical evidence of polyneuropathy and had PND scores of zero. Tendon xanthomas were observed in all patients except patient 1 (see Table 1).

**FIGURE 1:**
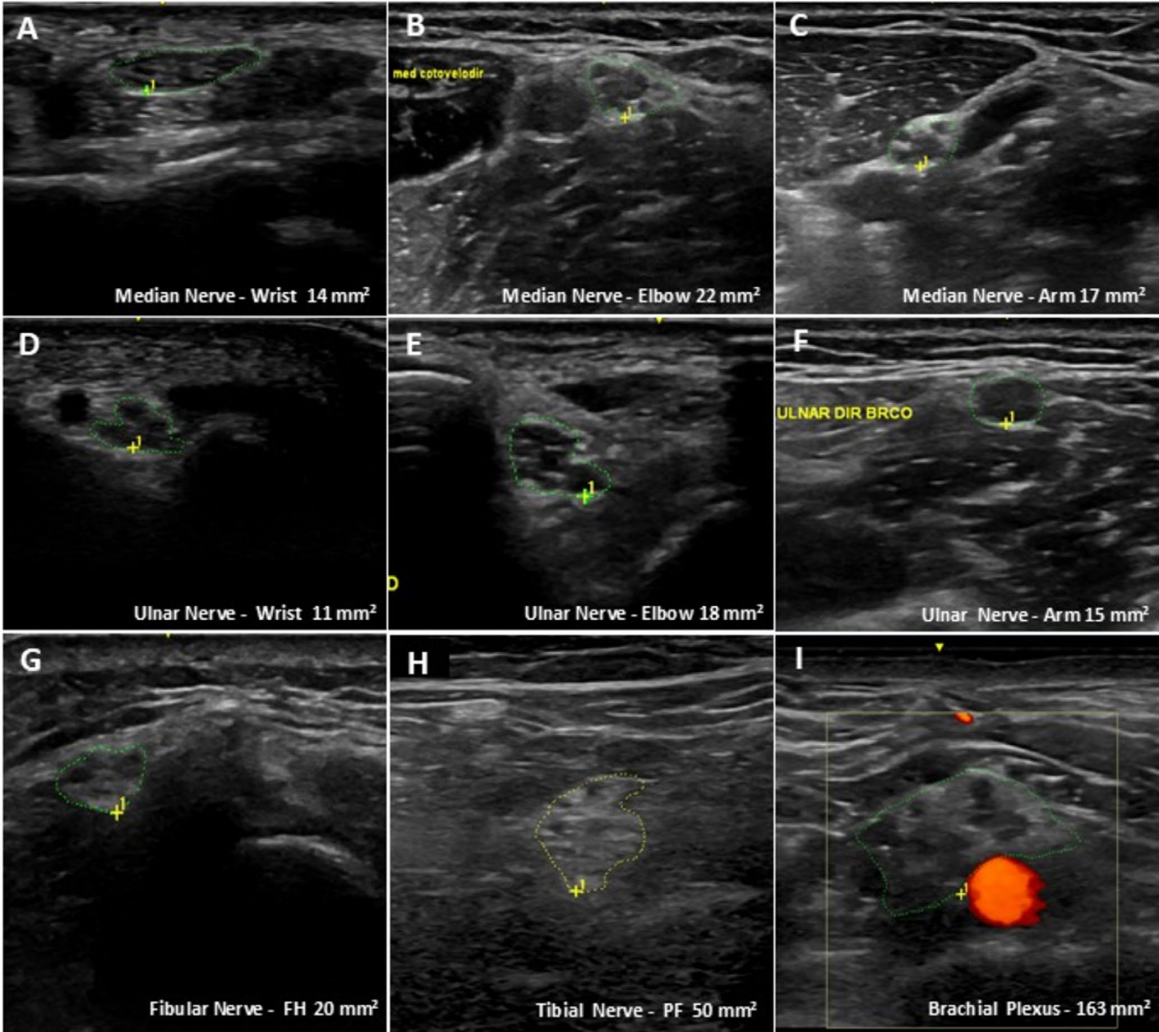
Nerve ultrasound findings in a patient with cerebrotendinous xanthomatosis. Representative ultrasound images demonstrating peripheral nerve enlargement in CTX—Patient 6. Color outlines (yellow and green) indicate the nerve boundaries used for cross‐sectional area (CSA) measurements (in mm^2^). Panels show: (A) Median nerve at the wrist (14 mm^2^), (B) Median nerve at the elbow (22 mm^2^), (C) Median nerve at the arm (17 mm^2^), (D) Ulnar nerve at the wrist (11 mm^2^), (E) Ulnar nerve at the elbow (18 mm^2^), (F) Ulnar nerve at the arm (15 mm^2^), (G) Fibular nerve at the fibular head (20 mm^2^), (H) Tibial nerve at the popliteal fossa (50 mm^2^), and (I) Brachial plexus at the supraclavicular level (163 mm^2^).

Nerve ultrasound findings are summarized in Table 2. Ultrasound revealed increased nerve CSA at multiple sites, both proximal and distal segments, across several nerves in the upper and lower limbs, with a proximal predilection, even in pre‐symptomatic patients with normal NCS (pts 1, 2, and 3) (Figure 1). All patients with CTX exhibited at least one enlarged peripheral nerve. Pt1, a 9‐year‐old child, showed isolated enlargement of the supraclavicular brachial plexus compared with the matched control. After excluding Pt1, analysis of the remaining five adult CTX patients (Pt2–Pt6) revealed widespread nerve CSA enlargement. At the supraclavicular brachial plexus, enlargement was present in all adult patients. In the upper limbs, the median nerve showed the most consistent abnormalities: enlargement was observed in 4/5 patients at both the wrist and the forearm, and in all patients at the cubital fossa and the upper arm. The ulnar nerve exhibited a more heterogeneous pattern, with enlargement most prominent in the upper arm (5/5). Lower‐limb involvement was also present, most notably with frequent enlargement of the fibular nerve at the fibular head (4/5), followed by the tibial nerve at the popliteal fossa (3/5), while sural nerve enlargement was uncommon (1/5).

**TABLE 2:** Cross‐sectional areas (CSA) of peripheral nerves in patients with cerebrotendinous xanthomatosis (CTX).

Site	Pt 1	C1	Pt 2	C2	Pt 3	C3	Pt 4	C4	Pt 5	C5	P 6	C6	Ref
SC Brachial plexus	57	35.4	**86**	41.1	**95**	63.9	**87**	56	**159**	82	**163**	53	≤ 82
Median wrist	4	5	**13**	5	8	7.5	**10.9**	5	**15**	8.2	**14**	8	≤ 10
Median forearm	3	3	**13**	3	6	5.6	**8.6**	4	**10**	6.1	**10**	5.1	≤ 9
Median elbow	4	4	**18**	6	**15**	8.5	**12.5**	7	**18**	8.5	**22**	7.9	≤ 12
Median arm	4	4	**15**	7	**13**	9	**15.2**	6	**13**	8	**17**	7.3	≤ 12
Ulnar wrist	3	2.5	7	4	4	6	3.5	5	4	4.7	**11**	4.5	≤ 9
Ulnar forearm	5	5	**11**	5	**9**	4.2	7	6	7	7	**11**	3.8	≤ 8
Ulnar elbow	3	4	**11**	6	5	5.5	7.5	4	**16**	6.7	**18**	6.2	≤ 10
Ulnar arm	3	3	**10**	5	**9**	6	**9**	4	**12**	6.4	**15**	7.2	≤ 8
Tibial ankle	6	5	9	7	9	6	5	6	4	9	8	10	≤ 12
Tibial PF	17	18	**42**	26	24	27	32	24	**59**	25.3	**50**	23.1	≤ 32
Fibular FH	6	5	**20**	7	8	6	**13**	7	**23.5**	12	**20**	8.5	≤ 12
Sural leg	2	2	3	2	1	2	1.5	2.6	2	3	**4.3**	2	≤ 3

*Note*: CSA values are expressed in mm^2^. Bold font indicates abnormal values.

Abbreviations: C, control; CSA, cross‐sectional area; CTX, cerebrotendinous xanthomatosis; FH, fibular head; PF, popliteal fossa; Pt, patient; Ref, Adult reference values (patients 2–6); SC, supraclavicular.

## 4 Discussion

This study demonstrated that nerve CSAs are enlarged in patients with CTX, with a predilection for proximal segments—most notably the supraclavicular brachial plexus and the proximal median nerve—and additional involvement of the ulnar, tibial, and fibular nerves. Notably, this enlargement was observed even in patients without clinical or electrophysiological evidence of polyneuropathy, suggesting that structural nerve changes may precede functional impairment. These findings suggest that ultrasound may serve as a valuable tool for early detection and clinical staging in this disorder.

Neuromuscular ultrasound (NMUS) has been proposed as a valuable tool for assessing peripheral nerve involvement in several ataxia–neuropathy syndromes [27]. Nerve ultrasound can help delineate disease‐specific peripheral nerve signatures, and depending on the underlying pathology, nerve CSA may appear normal, reduced, or enlarged [28]. A reduction in nerve CSA has been described as a characteristic feature of sensory ganglionopathy, reflecting axonal loss secondary to dorsal root ganglion degeneration [29]. This pattern is typically observed in syndromes such as cerebellar ataxia with neuropathy and vestibular areflexia syndrome (CANVAS) [30], spinocerebellar ataxia Type 2 (SCA2) [31], and ataxia–telangiectasia (A‐T) [31]. Conversely, disease‐specific patterns of nerve enlargement have been reported in patients with metabolic inborn errors, including X‐linked adrenoleukodystrophy (X‐ALD) [32] and metachromatic leukodystrophy (MLD) [33]. Interestingly, in autosomal recessive spastic ataxia of Charlevoix–Saguenay (ARSACS), nerve ultrasound studies have shown no significant nerve enlargement, even though the neuropathy is predominantly demyelinating [34].

The nerve ultrasound pattern observed in our cohort is consistent with the previously published CTX nerve ultrasound studies [20, 21]. CTX patients show a predominant enlargement of upper‐limb nerves, particularly the median nerve, which has been enlarged in all reported cases—4/5 at the wrist, 5/5 in the forearm, 5/5 at the cubital fossa, and 4/4 in the upper arm [20, 21]. The ulnar nerve shows a more variable pattern, with elbow enlargement being frequent (4/5 cases) [20, 21]. Lower‐limb involvement has also been reported, though less consistently. The tibial nerve was enlarged in 3/5 patients, both at the popliteal fossa and at the ankle. The fibular nerve demonstrated enlargement in 3/5 studies at the fibular head [20, 21]. Also, sural nerve enlargement has been identified in 2/3 examined patients [20]. Our findings align with prior literature [20, 21] and place CTX within the spectrum of inborn errors of metabolism that can lead to polyneuropathy characterized by multifocal nerve enlargement, with a possible proximal predilection in the upper limbs and brachial plexus.

Based on our findings and the existing literature [20, 21], we propose a practical ultrasound screening strategy to help distinguish CTX from other neuropathies—or at minimum to raise suspicion for CTX in patients with tendon xanthomas even before genetic confirmation. Initial assessment should include the median and ulnar nerves at the wrist, elbow, and arm, as well as the tibial nerve at the popliteal fossa and the fibular nerve at the fibular head, since these segments demonstrated the highest diagnostic yield in our cohort and in previous reports [20, 21]. When feasible, assessment of the supraclavicular brachial plexus or cervical roots may also provide additional information, although this requires further validation. To facilitate longitudinal monitoring, we emphasize the use of fixed anatomical landmarks, standardized machine settings, and consistent patient positioning, which enable reliable repeated measurements across follow‐up visits [35].

In our cohort, three patients had been on CDCA therapy for 2 years, which might have introduced bias into the NCS/EMG and ultrasound findings. However, given the cross‐sectional design and small sample size, no definitive conclusions can be drawn. Long‐term follow‐up of these patients is planned and will provide valuable insights into the evolution of peripheral nerve changes in CTX.

This was an exploratory study, and confirmation of these findings will require more extensive, multicenter investigations. Essential limitations include the relatively small sample size and the partial blinding of the ultrasonographer, limiting the generalizability of the results. A further limitation is that cervical root evaluation was not performed. Instead, proximal nerve involvement was assessed through supraclavicular brachial plexus CSA, which provided greater technical reproducibility for our sonographer [24] and was more feasible, as behavioral symptoms—particularly agitation—in some patients made detailed root scanning unreliable. Another limitation is the cross‐sectional design, which precluded longitudinal assessment and prevented evaluation of the role of ultrasound in disease monitoring. Furthermore, future studies incorporating magnetic resonance neurography (MRN) and tendon imaging assessments may provide additional insights into neuromuscular involvement in CTX.

Our results suggest that nerve ultrasound is a valuable complementary tool to standard NCS/EMG in assessing peripheral nerve involvement in CTX. We advocate for its integration into clinical practice as a non‐invasive method for detecting nerve enlargement, and future longitudinal studies are warranted to determine its potential role in monitoring disease progression in CTX.

## Author Contributions

**Antonio Edvan Camelo‐Filho:** investigation, conceptualization, writing – original draft, writing – review and editing, visualization, validation, methodology, software, formal analysis, project administration, resources. **Pedro Lucas G. S. B. Lima:** writing – original draft, investigation, conceptualization, writing – review and editing, methodology, software, formal analysis, validation, project administration. **Tito B. S. Soares:** conceptualization, investigation, writing – original draft, writing – review and editing, methodology, visualization, formal analysis, software. **Rodrigo Fagundes da Rosa:** writing – original draft, writing – review and editing, methodology, investigation, conceptualization, validation, visualization, software, formal analysis. **Luis Edmundo T. A. Furtado:** investigation, data curation, writing – review and editing. **Ana Lucila Moreira:** investigation, data curation, writing – review and editing. **André L. S. Pessoa:** conceptualization, investigation, writing – original draft, writing – review and editing, validation, software, formal analysis, supervision. **Paulo R. Nóbrega:** supervision, conceptualization, investigation, writing – original draft, writing – review and editing, visualization, validation, methodology, software, formal analysis, project administration. **Pedro Braga‐Neto:** supervision, data curation, conceptualization, investigation, writing – original draft, writing – review and editing, validation, methodology, formal analysis, project administration.

## Ethics Statement

We confirm that we have read the Journal's position on issues involved in ethical publication and affirm that this report is consistent with those guidelines.

## Conflicts of Interest

The authors declare no conflicts of interest.

## Supporting information


**Data S1:** NCS in CTX patients with polyneuropathy.

## Data Availability

The data that support the findings of this study are available on request from the corresponding author. The data are not publicly available due to privacy or ethical restrictions.
