# Supplementary material for: Nerve Ultrasound Detects Peripheral Nerve Enlargement in Cerebrotendinous Xanthomatosis
Source: Muscle Nerve. 2026 Mar 26;73(6):1082–8. doi: 10.1002/mus.70221 (PMC13138356; doi:10.1002/mus.70221)
Supplement: Supplementary file 1 — Data S1: NCS in CTX patients with polyneuropathy. [file MUS-73-1082-s001.docx]

Supplementary Material. NCS in CTX patients with polyneuropathy.

|  |  | Patient 4 | Patient 5 | Patient 6 |  |
| --- | --- | --- | --- | --- | --- |
| Height (cm) |  | 156 | 154 | 159 |  |
| Motor Conduction Studies:  DL;  CMAP amplitude;  MNCV; | Median  (Right/Left) | 4.1/4.0  11.5/9.4  54.4/51.2 | 3.2/3.5  6.2/11.5  **45.9**/52.5 | 3.5/3.8  14.1/12.3  **46.9/46.8** |  |
|  | Ulnar  (Right/Left) | 3.3/3.2  16.3/16.4  55.8/52.1 | 3.1/2.6  7.7/7.6  **48.4/47.8** | 2.7/2.8  15.6/14.6  51.1/**46.8** |  |
|  | Tibial  (Right/Left) | 4.2/4.1  19.2/16.7  **28.9/27.1** | 3.3/3.7  **0.5/0.8**  **29.5/20.8** | 3.2/3.3  12.8/11.4  **33.7/30.8** |  |
|  | Fibular  (Right/Left) | 4.9 / 4.3  6.8/5.7  **35.6/34.1** | 3.3/3.9  **0.37/0.42**  **28.3/31.1** | 3.7/4.4  3.6/3.3  **33.8/34.8** |  |
| F-wave (min latency) | Ulnar  (Right/Left)  Tibial  (Right/Left) | **30.1/30.4**  **63.1/64.2** | **29.2/30.1**  - / - | **29.5/28.9**  **55.3/57.6** |  |
| Sensory Conduction Studies:  SNAP amplitude;  SNCV | | Median  (Right/Left) | 66.2/55.9  50.6/52.3 | 35.7/37.7  57.4/58.8 | 20.1/21.6  **47.1/46.1** |
|  |  | Ulnar  (Right/Left) | 55.9/41.0  53.4/52.1 | 28.5/21.9  58.6/52.9 | **8.2**/**13.5**  **46.8/45.5** |
|  |  | Sural  (Right/Left) | 12.6/9.9  54.3/52.7 | **5.1/5.7**  49.9/54.3 | - / - |
| H-reflex (min latency) | Tibial  (Right/Left) | **30.7/31.1** | **31.7/32.1** | **32.7/32.5** |  |

Abbreviations: DL (Distal Latency), CMAP (Compound Muscle Action Potential, millivolts), MNCV (Motor Nerve Conduction Velocity, meters per second), SNAP (Sensory Nerve Action Potential, microvolts), and SNCV (Sensory Nerve Conduction Velocity, meters per second). "-": No potential evoked. Bold numbers indicate abnormalities.
